# Recent Advances in Barrier Layer of Cu Interconnects

**DOI:** 10.3390/ma13215049

**Published:** 2020-11-09

**Authors:** Zhi Li, Ye Tian, Chao Teng, Hai Cao

**Affiliations:** 1 Institute of Marine Biomedicine, Shenzhen Polytechnic, Shenzhen 518055, China; lizhireal@163.com; 2 State Key Laboratory of Materials-Oriented Chemical Engineering, College of Chemical Engineering, Nanjing Tech University, Nanjing 211816, China; 3 National Laboratory of Solid State Microstructures, College of Engineering and Applied Sciences, Nanjing University, Nanjing 210093, China; ytian@nju.edu.cn

**Keywords:** Cu diffusion barrier, platinum group metals, 2D materials, self-assembled monolayers, high entropy alloys

## Abstract

The barrier layer in Cu technology is essential to prevent Cu from diffusing into the dielectric layer at high temperatures; therefore, it must have a high stability and good adhesion to both Cu and the dielectric layer. In the past three decades, tantalum/tantalum nitride (Ta/TaN) has been widely used as an inter-layer to separate the dielectric layer and the Cu. However, to fulfill the demand for continuous down-scaling of the Cu technology node, traditional materials and technical processes are being challenged. Direct electrochemical deposition of Cu on top of Ta/TaN is not realistic, due to its high resistivity. Therefore, pre-deposition of a Cu seed layer by physical vapor deposition (PVD) or chemical vapor deposition (CVD) is necessary, but the non-uniformity of the Cu seed layer has a devastating effect on the defect-free fill of modern sub-20 or even sub-10 nm Cu technology nodes. New Cu diffusion barrier materials having ultra-thin size, high resistivity and stability are needed for the successful super-fill of trenches at the nanometer scale. In this review, we briefly summarize recent advances in the development of Cu diffusion-proof materials, including metals, metal alloys, self-assembled molecular layers (SAMs), two-dimensional (2D) materials and high-entropy alloys (HEAs). Also, challenges are highlighted and future research directions are suggested.

## 1. Introduction

Ever since the development of the integrated circuit (IC) about 60 years ago, aluminum (Al) and silicon dioxide (SiO_2_) have been most widely used as conductor and insulator materials for the fabrication of micro-processors [1,2]. As technical demands grew, the continuous decrease of the feature sizes and the explosive increase of the number of transistors in micro-processors resulted in the growth of so-called gate delays [3,4]. To solve this issue, new wiring materials with resistivity lower than Al and dielectric materials with dielectric constant (so-called low-κ) lower than conventional SiO_2_ have to be used as alternatives. IBM announced in 1997 the replacement of Al with copper (Cu) as the interconnect material in semiconductor processing [5]. As compared to Al, Cu has a smaller gate delay due to its lower electrical resistivity, but higher electro-migration, stress-migration resistances and melting point [6]. However, a big problem of switching Al to Cu is that the conventional methods used for Al deposition (sputter deposition) and patterning (reactive ion etching) are not suitable for Cu, as Cu is corroded during standard chip manufacturing processes. Therefore, the fabrication technique has to be upgraded for Cu patterning and deposition.

The structure of Cu interconnects is usually patterned by a so-called damascene process [7,8], in which the dielectric layer is patterned in advance, followed by the sequential deposition of a diffusion barrier layer and the filling of the patterned trenches with Cu. The excess of Cu can be removed by a chemical mechanical polishing (CMP) process. Among the many deposition methods of Cu, the electrochemical deposition (ED) technique [9,10] has been proven to be the most economic and efficient way to super-fill damascene features without defects, as compared to techniques such as electroless plating, vacuum-based physical vapor deposition (PVD) and chemical vapor deposition (CVD). The atomic layer deposition (ALD) method is another way to generate uniform Cu thin film. Based on sequential layer-by-layer deposition and self-limiting behavior, ALD provides high conformity of thin film quality and accurate control of layer thickness, in spite of the slow deposition rate and low throughput. Inspired by this method, electrochemical atomic layer-by-layer deposition (known as *e*-ALD) has been developed to fabricate ultra-thin Cu film, including two main steps: (1) deposit a sacrificial atomic layer of an appropriate metal by holding an electrode potential within the underpotential deposition region; (2) release the electrode potential to induce the spontaneous displacement of sacrificial metal layer by atomic Cu layer [11]. However, the introduction of copper as interconnects also raises some other challenges, including the degradation of devices due to the diffusion of Cu into the Si and Si-based insulating layers at rather low temperatures [12], the absence of a self-passivized oxide layer causing the corrosion of Cu under chip fabrication process, as well as the poor adhesion between Cu and insulating layers.

To solve these problems, a suitable barrier material with good adhesion to Cu is required to prevent Cu from diffusing into the dielectric layer. The qualified diffusion barrier materials need to be refractory and inactive to both conductors and insulators at rather high temperatures, normally including transition metals such as tantalum (Ta) [13,14,15], tungsten (W) [16,17,18], titanium (Ti) [19,20] and their composites with nitrogen (N), carbon (C) or Si, such as Ta/TaN [21,22,23,24,25], W_2_N [26,27,28], TiN [29,30,31,32], TiC [33,34,35], TaSiN [36,37,38], Si_3_N_4_ [39] and so on. As those state-of-the-art barrier materials are typically poorly conductive, pre-deposition of a Cu seed layer is often needed for the electroplating of Cu, but the Cu seed layer is prone to dissolution in an acidic electrolyte in the subsequent ED process, making it hard to obtain a uniform Cu layer. However, direct plating of uniform Cu film on diffusion barrier materials is of crucial importance in the modern fabrication process. This review briefly summaries the latest development in Cu barrier materials, including state-of-art Ta/TaN, platinum group metals (PGMs) such as ruthenium (Ru)-based materials, 2D materials, self-assembled molecular layers (SAMs) and high-entropy alloys (HEAs). Some of those new barrier materials provide not only reliable Cu diffusion barrier properties during thermal annealing, but also anti-corrosion of Cu in the electrolyte. High-quality ultra-thin film of CVD graphene [40], hexagonal boron nitride (h-BN) [41], magnetron sputtering HEAs [42] and dip-coated SAMs [43] have shown great ability to prevent metals from corrosion in salty solution. In addition, some metal oxide layers (e.g., Ru oxide [44] and Ir oxide [45]) have also been proven as reliable metal corrosion resistants.

## 2. Cu Interconnects and Diffusion Barrier Materials

**Figure 1:**
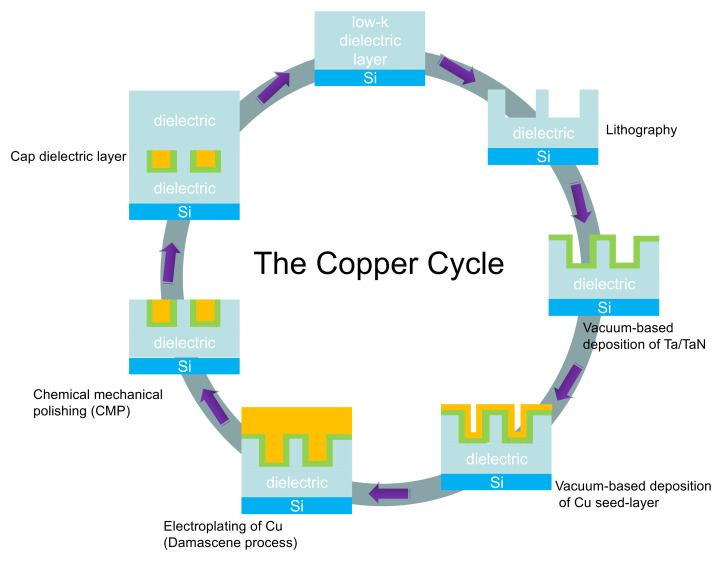
Schematic demonstration of the Cu cycle.

Cu interconnects function as internal wiring, connect each circuit compartment and distribute power. In the damascene process, the Cu wiring technique can be vividly demonstrated by the so-called Cu cycle [46] depicted in Figure 1, which combines a series of individual processing steps to fabricate a single level of Cu interconnect architecture on a Si wafer.

Typically, the Cu cycle starts from the deposition of a low-κ dielectric layer on the Si wafer. Afterwards, the dielectric film is patterned by lithographic method. Then a thin film of Ta/TaN diffusion barrier layer is deposited on top of the dielectric pattern by means of PVD or CVD. Due to the low conductivity and poor nucleation behavior of Cu on the Ta/TaN layer, vacuum deposition of Cu seed-layer is needed to ensure that the following ED process of Cu is able to superfill the damascene features. Finally, a CMP process is conducted to remove the over-plated Cu. The Cu cycle restarts with the deposition of another dielectric layer. During the whole cycle, the defect-free filling of Cu in the damascene trenches is crucial.

**Figure 2:**
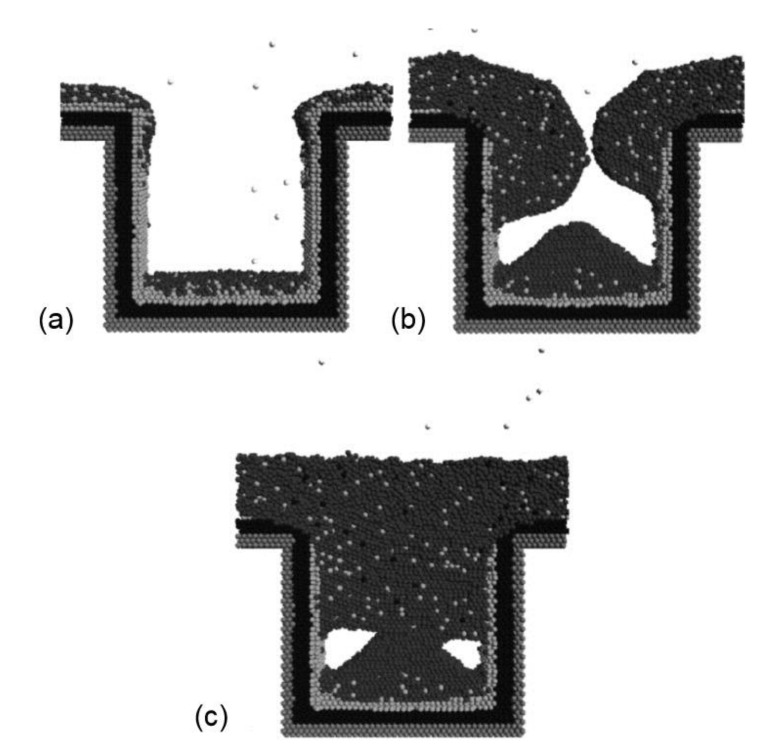
Schematic illustration of the “over-hang” formed by electroless deposition of Cu seed layer, leading to the voids formed by the subsequent electrochemical Cu deposition during a damascene process. (**a**) The formation of Cu overhanging clusters via electroless deposition; (**b**) growth of Cu overhanging clusters during electrochemical Cu deposition; (**c**) failure of Cu super-filling of damascene feature. Reproduced from Hong et al. [79]. Copyright 2005 Elsevier Ltd. All rights reserved.

However, according to Moore’s law [47], the number of transistors in a chip doubles every two years, that is, next generation devices demand the continuous decrease of feature sizes, which as a consequence increases the difficulty of defect-free filling of the trenches in the damascene process. It is well known that the resistances of conventional diffusion barrier materials are too high to be the substrate for direct ED of Cu [48,49,50,51], and an unpleasant phenomenon called “terminal effect” often appears [11,52,53,54,55,56,57,58,59]. This effect becomes more pronounced with the transition from 200 to 300 mm Si wafer. Normally the electrical contact is placed at the periphery of the wafer. When the ED of Cu is performed on a resistive substrate, there is a dramatic IR drop (potential gradient) across the wafer from the contact point to the wafer center, resulting in the non-uniform distribution of current over the resistive substrate with inhomogeneous Cu ED deposition. For this reason, an extra Cu seed layer has to be deposited in advance via PVD [60,61,62,63], CVD [64,65,66,67,68,69], ALD [70,71,72,73] or electroless methods [74,75,76,77,78]. The seed layer prepared by some methods such as PVD or electroless deposition normally causes undesired “over-hang” at the trench opening, which becomes devastating within the sub-45 nm region and results in unsuccessful filling in the following Cu ED process, as illustrated in Figure 2 [79].

To avoid this, new barrier materials and techniques must be developed. Desirable characteristics for ideal diffusion barrier materials have been proposed [80,81], including (1) excellent adhesion to Cu metal and dielectric layer; (2) immiscibility with Cu and an ability to prevent Cu diffusion at high temperatures; (3) good conductivity for direct ED of Cu; and (4) simplicity of uniform deposition of ultra-thin film on dielectric layer. In order to find a suitable replacement of traditional barrier materials, attention has been paid to PGM-based materials (e.g., Ru [82,83,84,85], iridium Ir [86,87,88,89], palladium Pd [90] and their composites with other materials [91,92,93,94,95,96,97]), 2D materials (e.g., graphene [98,99], hexagonal boron nitride h-BN [100], and molybdenum disulfide MoS_2_ [101,102]), SAMs [103,104,105,106,107] and HEAs [108,109,110,111]. The comparison of properties between new barrier materials and traditional Ta/TaN is listed in Table 1. Compared to Ta/TaN, PGMs (e.g., Ru and Ir) and 2D materials (e.g., graphene) have lower electrical resistivity and comparable melting point. The SAMs’ electrical resistivity and melting point strongly depend on their molecular nature, while HEAs have a poor electrical resistivity and their melting point is normally over 1000 °C. SAMs have the easiest deposition method by immersing substrate into the solution containing appropriate molecules. Concerning the layer thickness, 2D materials hold great potential for size downscaling, as single-layer graphene is only one-atom thick.

## 3. Platinum Group Metals (PGM)-Based Materials

**Figure 3:**
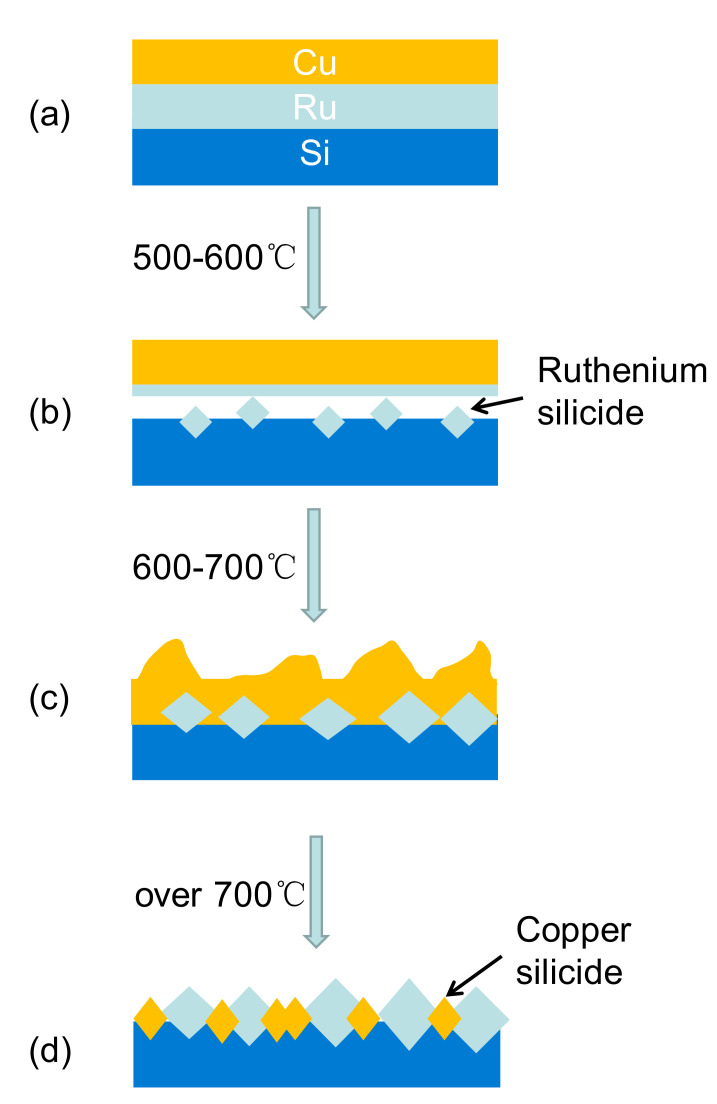
Schematic description of the barrier failure mechanism in Cu/Ru/Si system. (**a**) Intact Ru barrier at the initial stage, (**b**) barrier failure induced by the formation of ruthenium silicide, (**c**) complete dissolution of metallic Ru to form ruthenium silicide, and (**d**) Cu diffusion through ruthenium silicide to form copper silicide.

Among PGM metals, Ru receives the most attention. It is an air-stable metal with a much lower electrical resistivity (ρ_Ru_ = 7.1 μΩ·cm) [83] compared to that of Ta (ρ_Ta_= 13 μΩ·cm), which allows the direct electrochemical plating of Cu. More importantly, Ru has a melting point as high as 2334 °C [112], shows negligible solubility [113,114,115] but fantastic wettability with Cu and exhibits excellent adhesion to electroplated Cu at elevated temperatures [82,83,84,85]. Therefore, Ru has been considered as a promising candidate to replace the traditional diffusion barrier materials. Thin films of a Ru barrier layer can be placed on solid substrate via gas phase deposition methods such as PVD, CVD and ALD, or wet deposition methods such as ED and electroless plating. Chyan et al. [84] showed a successful example of the direct ED of a conformal Cu coating layer with controllable thickness on polycrystalline Ru electrode. Annealing up to 600 °C caused no apparent dewetting at the Cu/Ru interface, and more importantly, there was no new phase formed upon further annealing at 800 °C. Chan et al. [85] demonstrated that a high-quality thin film of Cu layer can be formed on top of a 20 nm thin film of Ru deposited on a Si wafer via a standard magnetron sputtering system, showing a Cu ED efficiency as high as 95%. The 20 nm Ru film was sufficient to prevent Cu from diffusing into Si upon annealing at 450 °C for 10 min, but the delamination of Ru thin film from a Si wafer can be seen at 550 °C, resulting in the penetration of Cu into Si substrate. However, reduction of the thickness of Ru layer leads to a decrease in both the Cu ED efficiency and the Cu diffusion-proof temperature. Arunagiri et al. [83] showed that even though a Ru thin film with reduced thickness of 5 nm was able to arrest the diffusion of Cu into Si after annealing at 300 °C for 10 min, only a Cu ED efficiency around 90% was obtained, and a new ruthenium silicide phase was formed at the temperature of 450 °C. Accordingly, a failure mechanism of Cu/Ru/Si system (Figure 3) was proposed by Damayanti et al. [116]. They suggested that the failure appeared at high annealing temperatures and was initiated by the formation of polycrystalline ruthenium silicide, further promoting the diffusion of Cu into Si substrates with the formation of copper silicide protrusions.

In a way, the quality of Ru thin film has a decisive influence upon the Cu interdiffusion behavior. The presence of Ru grain boundaries work as defective sites and greatly accelerate Cu interdiffusion through the Ru barrier layer. Chan et al. [85] provided evidence that the failure of using a thin Ru film as Cu diffusion barrier layer was caused by its columnar grain structure, which promoted the penetration of Cu atoms into the Si substrate at elevated temperatures. Their cross-sectional transmission electron microscopy (TEM) results clearly show columnar microstructures of a thin film of Ru oriented vertically on the Si substrate. Upon annealing at 550 °C, there was the detachment of Ru thin film from the Si substrate and the penetration of Cu through the Ru columnar grain boundaries into the Si underneath, resulting in the degradation of the Cu/Ru/Si system. However, the introduction of foreign substances into Ru thin film can cause the formation of amorphous structures, thereby eliminating the formation of columnar structure, controlling the grain boundaries and eventually improving the Cu interdiffusion resistance. Damayanti et al. [117] proved that the dissolution of nitrogen into a Ru thin film created an amorphous structure with a 10 times higher sheet resistance, and the doping delayed the formation of ruthenium silicide and reduced the Cu diffusion into the dielectric layer.

**Figure 4:**
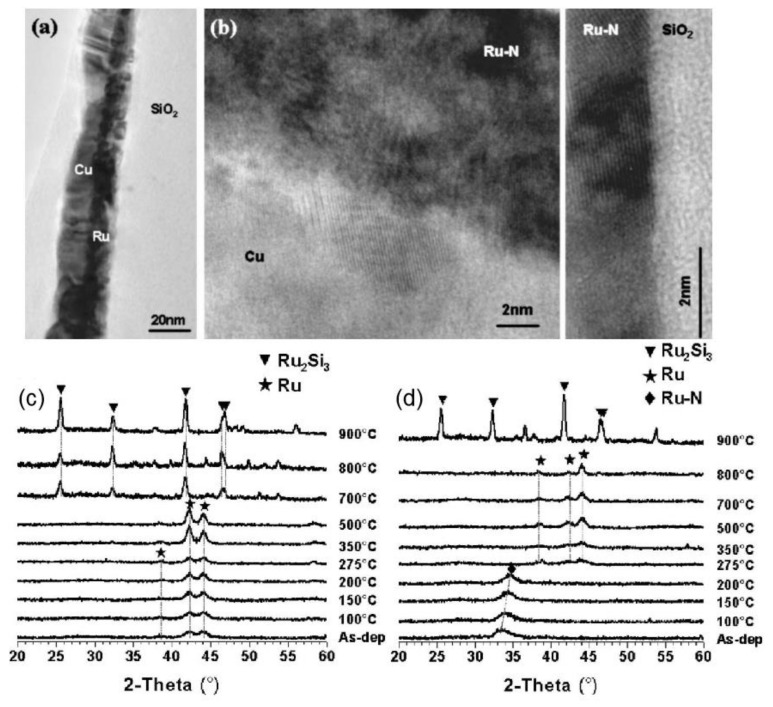
Cross-sectional transmission electron microscopy (TEM) micrographs of (**a**) as-deposited Cu/Ru/SiO_2_ and (**b**) Cu/Ru-N/SiO_2_ samples. X-ray diffraction (XRD) spectra of (**c**) Cu/Ru/Si and (**d**) Cu/Ru-N/Si at different annealing temperatures. Adapted from Damayanti et al. [117]. Copyright 2006 American Institute of Physics.

Figure 4 shows a comparison between the polycrystalline Ru and the amorphous Ru-N thin films prepared by a sputtering deposition method based on the TEM images and X-ray diffraction (XRD) spectra. The Ru barrier sputtered in Ar was in the form of polycrystalline with columnar structures (Figure 4a). In contrast, the sputtered Ru in nitrogen atmosphere arranged into amorphous structure, as nitrogen atoms occupied the interstitial sites and disrupted the crystallization of Ru. Upon annealing, nitrogen atoms were released from the Ru-N lattice and Ru atoms rearranged into a hexagonal structure. It has been suggested that a large number of nitrogen atoms got into the grain boundaries during the recrystallization of Ru crystal, serving as a “grain boundary stuffing” to prevent Cu diffusion. X-ray diffraction (XRD) results supported the proposition. Figure 4c shows the XRD spectra of Ru/Si system at different annealing temperatures. Peaks of crystalline Ru became narrower and more pronounced when the annealing temperature arose from 500 °C to 700 °C, likely caused by the gradual growth of nanocrystalline Ru grain at higher temperatures. When the temperature was above 700 °C, peaks of Ru_2_Si_3_ appeared, along with the gradual disappearance of peaks of crystalline pure Ru. Some distinct behaviors in the XRD spectra of the Ru-N/Si system were revealed, as shown in Figure 4d. A single broad peak at 2-Theta = 33.5° (marked by rhomboid) was present in the as-deposited Ru-N/Si sample. It was attributed to the formation of embryonic Ru-N clusters and moved to a higher 2-Theta number upon annealing at 200 °C due to the stress relaxation. It was worth noticing that the decomposition of Ru-N and the rearrangement of Ru atoms started at 275 °C. From 275 °C to 800 °C, the absence of an Ru-N peak and existence and growth of Ru peaks can be identified. While Ru_2_Si_3_ was already formed in Ru/Si system at 700 °C, the failure temperature of Ru-N/Si system was up to 900 °C.

Many studies [118,119,120,121,122,123,124] have demonstrated that some foreign elements, such as boron (B), phosphorus (P) and carbon (C) are able to induce the formation of amorphous Ru thin films and thereby significantly improve the ability of Ru thin films to prevent Cu interdiffusion. A 12 nm thin film of Ru (P) deposited on a low-*κ* dielectric layer effectively prevented the diffusion of Cu into Si wafer at 800 °C for 5 min [124]. Perng et al. [123] proved that the temperature of forming an amorphous phase of a 5 nm thin film of Ru-B-C was significantly higher than that of pure Ru under thermal annealing and the doped film was thermally stable up to 750 °C. Metal elements can also be applied as dopant to form an amorphous structure with Ru and increase its thermal stability. Chen et al. [125] showed that a 15 nm amorphous Ru-Ta thin film was able to resist Cu interdiffusion at 700 °C for 30 min. Yeh et al. [126] used a 10 nm thin film of amorphous RuW as the seedless Cu diffusion barrier, of which the failure temperature can be as high as 700 °C. Hsu et al. [127] verified that a 5 nm ultrathin film of RuCr can still function as a seedless Cu diffusion barrier after annealing at 650 °C for 30 min. In addition to doping with metallic and non-metallic elements, the incorporation of Ru with traditional barrier materials to form multilayered structures also worked efficiently as direct-electroplating barrier layers. It was proved by Sari et al. [128] that a stacked layer of Ru (7 nm)/ WN_x_ (8 nm) was able to prevent Cu from diffusion into Si upon annealing at 750 °C for 30 min, whereas a 15 nm Ru thin film failed to prevent the formation of Cu3Si at 450 °C. With the help of a plasma-enhanced ALD technique, Kim et al. [129] incorporated Ru with TaN to form an amorphous Ru-TaN barrier layer, and showed that a 10 nm thin Ru-TaN film of can resist Cu diffusion at 700 °C for 30 min. Kim et al. [130] further showed that a 4 nm Ru layer deposited on a 2 nm TaCN thin film exhibits excellent Cu diffusion-proof ability while annealing at 550 °C for 30 min. In addition, the Ru oxide layer is a well-known diffusion barrier layer for metal anti-corrosion. The thin film of RuO_2_ can be prepared via cost-effective methods such as sol-gel, spin-coating and dip-coating approaches. However, due to its high resistivity (ρ_RuO2_ = 35.2 μΩ·cm), a RuO_2_ layer would arrest further direct electroplating of Cu. Furthermore, a RuO_2_ layer weakens the adhesion of Cu and the substrate, and that lowers the nucleation density of Cu [131]. Therefore, a thin film of metal Ru, rather than its oxide, is preferred as a barrier layer for direct Cu electroplating.

## 4. Two-Dimensional (2D) Materials

Since the discovery of graphene in 2004 [132], this one-atom-thick carbon film has gained tremendous attention due to its outstanding mechanical [133], thermal and electronic properties [134,135]. Applying graphene as new barriers is of particular interest because its compact 2D structure is impermeable to any metal atoms, and it is highly thermally and electrically conductive. Of equal importance is its extraordinary thermal and chemical stability. Graphene has been proven to be a reliable barrier material for preventing Cu diffusion [136,137]. Bong et al. [138] tested the ability of using CVD-grown graphene, graphene oxide (GO) and reduced graphene oxide (rGO) as Cu diffusion barriers. CVD-grown graphene showed the best Cu diffusion barrier performance. Similar to Ru barrier layer, the grain boundary size had a big impact on the performance of a graphene-based diffusion barrier layer, which was demonstrated by Roy et al. [139]. Engineering graphene grain size has been considered crucial for graphene-based barriers.

**Figure 5:**
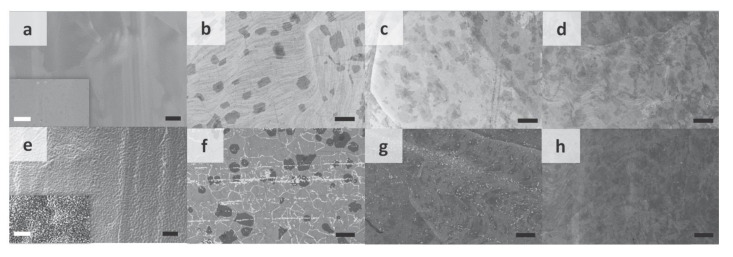
Scanning electron microscope (SEM) images comparing bare Cu, SLGx1_Cu (samples covered with layer of graphene), SLGx2_Cu, and SLGx4_Cu before (**a**–**d**, respectively) and after (**e**–**h**, respectively) 240 min of annealing in air at 200 °C. Scale bars = 10 μ m. Insets in (**a**) and (**e**) depict SEM images of wider areas. Scale bars = 1 μm. Reproduced from Roy et al. [139]. Copyright 2013 WILEY-VCH Verlag GmbH and Co. KGaA, Weinheim.

Figure 5 shows the scanning electron microscopy (SEM) images of bare Cu thin film and single- or multi-layer graphene-covered Cu taken before and after annealing in air at 200 °C for 240 min. Before annealing, bare Cu (Figure 5a) appeared as a smooth and polycrystalline thin film. Cu samples covered with one, two and four layers of graphene were named SLGx1_Cu (Figure 5b), SLGx2_Cu (Figure 5c) and SLGx4_Cu (Figure 5d), respectively. Cu_2_O was formed upon the annealing of Cu in air at 200 °C for 240 min, manifested as a rather rough surface revealed by SEM (Figure 5e). In comparison, significantly less oxidation or perturbation was observed on the surface of single-layer graphene-covered Cu, and changes only appeared at the grain boundaries and defective areas (Figure 5f). Even less oxidation was formed on SLGx2_Cu (Figure 5g) and barely any oxidation or perturbation were revealed on the SLGx4_Cu surface after annealing (Figure 5h). These results suggest that number of layers and grain size of graphene can effectively affect its barrier properties.

**Figure 6:**
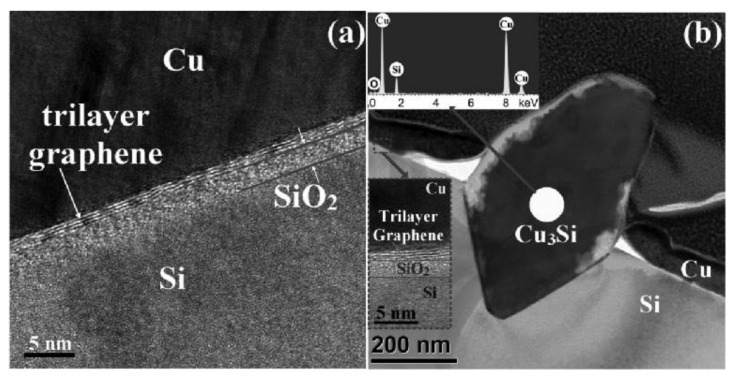
Cross-sectional TEM images of a tri-layer graphene film (**a**) before and (**b**) after 30 min of annealing at 700 °C. A distinct boundary between Cu and graphene can be seen even after (**b**) 5 min of annealing at 800 °C. Inset at top left in panel (**b**) indicates the formation of copper silicide and a cross-sectional image of the undegraded graphene layer is displayed at the bottom left. Reproduced from Nguyen et al. [140]. Copyright 2014 AIP Publishing LLC.

Nguyen et al. [140] showed that a Cu diffusion barrier made up of 1 nm-thick graphene tri-layer was thermally stable up to 700 °C for 30 min. The degradation started at 750 °C in 5 min whereas the breakdown occurred at 800 °C in 5 min. Figure 6a clearly showed that the trilayer graphene remained intact after annealing at 700 °C for 30 min and there was no apparent penetration of Cu into SiO_2_ or Si layers. In contrast, the formation of Cu_3_Si can be identified from Figure 6b, due to the structure damage of trilayer graphene at 800 °C for 5 min followed by Cu diffusion through the defects. In addition, Hong et al. [141] managed to improve the Cu diffusion barrier ability by engineering the grain size of single layer graphene. Their experimental results confirmed that single-layer graphene with average grain size of 2 ± 1 µm was thermally stable after annealing at 700 °C for 30 min whereas single-layer graphene with average grain size of 10 ± 2 µm was thermally stable after annealing at 900 °C for 30 min.

2D materials such as h-BN and transition metal dichalcogenides (TMDs) have also been explored as efficient Cu diffusion barriers. Liu et al. [142] showed that a few h-BN layers deposited by CVD on various metal substrates were impervious to oxygen diffusion even at high temperatures. Shen et al. [143] and Ren et al. [144] further confirmed that ultra-thin layer of h-BN can serve as a long-term oxidation-proof barrier for Cu due to its high impermeability and insulating characteristics. Furthermore, Lo et al. [145] showed the feasibility of using h-BN thin film as Cu diffusion barrier layer to block Cu penetration into dielectric layer. However, a drawback of applying h-BN as a Cu diffusion barrier is its resistive nature, arresting the direct ED of Cu on top. Furthermore, Mertens [146] showed that Cu intercalation into h-BN may occur through the defective areas during the ED process, leading to the delamination of h-BN layer. 2D TMDs such as molybdenum disulfide (MoS_2_), tungsten disulfide (WS_2_), molybdenum diselenide (MoSe_2_) and tungsten diselenide (WSe_2_) have also been proven to be potential candidates for the replacement of the traditional barrier [147]. Lo et al. [148] showed that single-layer MoS_2_ can be directly grown on dielectric layer by metal organic CVD with the size of high-quality layer achieved up to 1 cm^2^. Their results proved that single-layer MoS_2_ can effectively suppress Cu diffusion and dramatically increase the lifetime of a dielectric layer.

## 5. Self-Assembled Molecular Layers (SAMs)

Self-assembled monolayers with long-range order are formed by the spontaneous organization of molecules under thermodynamic or kinetic control. As a replacement for traditional barrier materials, it has been shown by Mikami et al. [149] that the lifetime of 1.7 nm-thick P containing SAM diffusion barrier is comparable to that of a 20 nm-thick Ta film. A self-assembled monolayer formed by P containing molecules showed excellent barrier performance owing to the formation of a Cu–P complex during annealing. Yoshino et al. [150] compared the barrier performance of SAM-modified SiO_2_ samples prepared with P, C and N containing molecules with an identical chemical structure. Barrier effect was observed by time-dependent dielectric breakdown measurements, suggesting that the time-to-breakdown of P-SAM modified SiO_2_ was 10 times longer than that of bare SiO_2_ while C-SAM and N-SAM showed inconspicuous improvements. It has been proved that the nature of SAMs’ terminal groups can significantly influence the barrier effect against Cu diffusion [151,152]. Caro et al. [153] evaluated a long list of SAMs with different head groups, chain lengths and terminal groups and concluded that -NH_2_ terminated SAM is the most promising barrier material for Cu diffusion.

**Figure 7:**
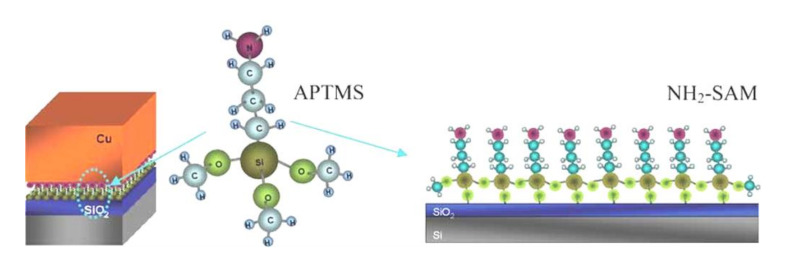
A schematic drawing of NH_2_-SAM (self-assembled molecular layer) barrier layer located in between Cu and SiO_2_. Reproduced from Caro et al. [106]. Copyright 2010 WILEY-VCH Verlag GmbH and Co. KGaA, Weinheim.

Caro et al. [106] further built up a Cu/NH_2_-SAM/SiO_2_ system by immersing pre-cleaned SiO_2_ into toluene solution of 3-aminopropyltrimethoxysilane to prepare sub-nm NH_2_-SAM as Cu diffusion barrier (Figure 7) and they found no evidence of Cu penetration through NH_2_-SAM up to 400 °C. However, failure occurred upon further annealing due to the weakened adhesion at the Cu/NH_2_-SAM interface at higher temperatures. To enhance the adhesion of NH_2_-SAM with Cu, Chung et al. [154] prepared a cleaned SiO_2_/Si substrate with (3-aminopropyl) trimethoxysilane to form a -NH_2_ terminated surface and then let it react with 3-mercaptopropionic acid to form a -SH terminal group on the top of NH_2_-SAM. The coupled SAM showed a significant increase of about 33% on the load force and interfacial adhesion energy.

Sharma et al. [155] modified the preparation procedure of NH_2_-SAM by immersing hydroxyl-terminated SiO_2_ into a toluene solution of 3-aminopropyltrimethoxysilane in the presence of a trace amount of water. 3-Aminopropyltrimethoxysilane molecules turned into hydroxyl-terminated Si through hydrolysis, which strongly bonded with hydroxyl-terminated SiO_2_ and nearby molecules via dehydration to form a stable monolayer. The Cu/NH_2_-SAM/SiO_2_ was functional as a barrier layer up to 600 °C.

## 6. High-Entropy Alloys (HEAs)

The concept of HEAs were first reported by Yeh in 2004 [156]. This new type of material is normally formed by mixing multi-principal (more than five) metallic elements in equal molar ratio. HEAs have been fabricated by physical and chemical methods [157], including a magnetron sputtering method [158,159,160], laser cladding [161,162], electrochemical deposition [163], electron beam evaporation deposition [164], and so on. Among those methods, the magnetron sputtering and laser cladding are the most widely used fabrication methods. HEAs show excellent properties such as high thermal stability, resistance to interdiffusion, hardness and chemical stability, thanks to their widely-known high-entropy, sluggish diffusion, lattice distortion and “cocktail” effects. The high entropy is the most important feature of HEAs, referring to that the solid-solution structure is more favorable than intermetallic compound due to the increased configurational entropy [165,166] in alloys with 5 or more elements. The sluggish diffusion effect suggests that the diffusion in HEAs is much slower than that in conventional alloys, which explains the high thermal and mechanical stability of HEAs [167,168,169]. Because HEAs are formed by mixing various elements, lattice distortion is unavoidably caused. Many researchers have observed the lattice distortion with the assistance of high-resolution TEM [170,171] and XRD [172]. Normally, the lattice strain is proportional to the misfit parameter of atomic size difference in HEAs, increasing their strength and hardness [173,174]. HEAs are defined by the componential elements, but mixing them often gives unique and unexpected properties, known as the “cocktail” effect first proposed in 2003 by S. Ranganathan [175]. These merits make HEAs a potential candidate of barrier material.

It is well known that the main challenges in using conventional metallic materials as a diffusion barrier are: (1) the Cu diffusion through the grain boundaries of metallic barriers, (2) the reaction between metallic barriers and Si [176], and (3) the chemical stability of doped metallic barrier is often insufficient to avoid the reaction with Si after annealing [176]. In contrast, previous studies clearly indicated that HEAs can easily form solid-solution or amorphous structures to suppress Cu interdiffusion, oxide film nucleation and growth, owing to the high configurational entropy and large lattice distortion caused by the different sizes of mixed atoms [177,178,179].

The lattice parameters and corresponding properties can be easily tuned by altering the molar ratio of the mixed elements. Tung et al. [180] synthesized 7 different AlCoCrCuFeNi high-entropy alloys via melting and casting method and demonstrated that the transformation of type of microstructure and the lattice constant resulted in changes of the materials’ hardness, as shown in Table 2 [180]. Reducing the content of the Al or Cr element significantly decreased the hardness of the HEAs, because the body-centered cubic (BCC) phase (enhanced by Al and Cr elements in the system) is much harder than the face-centered cubic (FCC) phase (enhanced by Co, Cu, Fe and Ni elements), according to the basic structure factor and solution-hardening mechanism, which was further confirmed by Tong et al. [181]. Interestingly, the introduction of nitrogen element into HEAs can also effectively improve HEAs’ hardness and Young’s modulus by filling the interstitial positions of the HEAs’ lattice [163], and can also suppress the crystalline structure formation [182].

**Figure 8:**
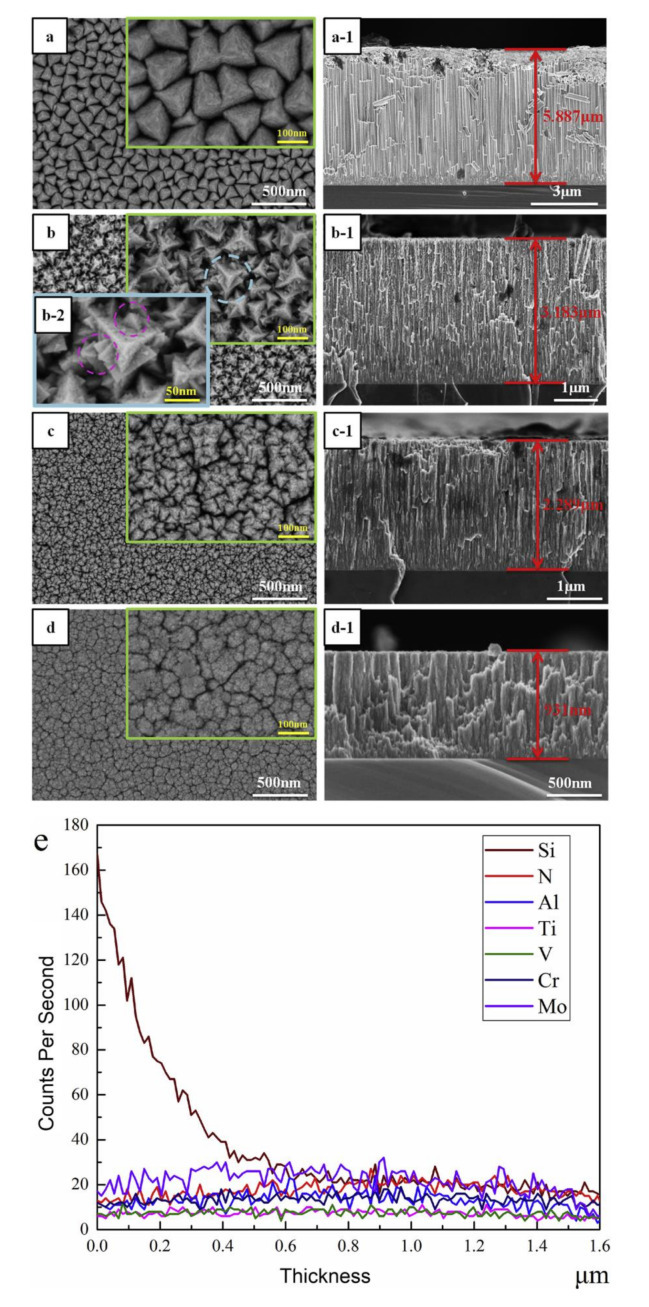
SEM micrographs of surface and cross-section morphologies of (**a**) VAlTiCrMo, (**b**) (VAlTiCrMo)N_x_-100, (**c**) (VAlTiCrMo)N_x_-450 and (**d**) (VAlTiCrMo)N_x_-800 coatings, (**e**) the energy-dispersive spectrometry scanning of element distribution along the thickness direction of the (VAlTiCrMo)N_x_-800 coating. Reproduced from Chen et al. [183]. Copyright 2020 Elsevier B.V. All rights reserved.

Chen et al. [183] investigated the effect of nitrogen content on the morphology and property of HEA-N. It can be seen from the SEM images shown in Figure 8a, a-1 that the N-free VAlTiCrMo HEA arranged into regular tetrahedral columnar structures with an average width over 100 nm perpendicular to the substrate. The surface morphology changed dramatically upon the introduction of nitrogen with a flow rate of 100 standard cubic centimeters per minute (sccm). The gaps between tetrahedral columns were filled by many small particles to form a more compact structure, and the density of barrier layer arises in response to the increasing of content of nitrogen, as shown in Figure 8b–d. The Figure 8e showed an uniform element distribution along the thickness direction of the (VAlTiCrMo)N_x_-800 barrier layer, provided by the energy-dispersive spectrometry.

Jiang et al. [184] tested the feasibility of using HEA-N as barrier to prevent Cu diffusion at high temperatures. Nitrogen atoms were introduced into AlCrTaTiZr HEA lattice under content control, in order to prepare AlCrTaTiZr/AlCrTaTiZr-N composite. The diffusion barrier performance of such material was tested under thermal annealing up to 900 °C and no Cu–Si intermetallic compounds were generated. The barrier property can be attributed to the amorphous structure of AlCrTaTiZr layer and increased Cu diffusion distance caused by the lattice mismatch between AlCrTaTiZr and AlCrTaTiZr-N layers.

## 7. Conclusions and Perspectives

The “revolution” ignited by the introduction of Cu interconnects has boosted the development of the IC industry. As the technical demands grow, reliable Cu diffusion barrier materials should play significant roles not only in physical separation between Cu and the dielectric layer, but also in the prevention of Cu diffusion at high temperatures while downscaling feature size. As discussed in depth above, potential barrier materials such as Ru-based materials, 2D materials, SAMs and HEAs have proven their great ability to prevent Cu from diffusing even at few nm thickness, which is essential for continuous downsizing of transistors. Ultra-thin film of Ru is conductive enough for direct ED of Cu in a damasence process and has a good barrier effect against Cu diffusion, whereas the quality of Ru thin films is of crucial importance for their application as barriers to prevent Cu penetration at high temperatures, and that integrating Ru with foreign elements can bring significant changes to its crystalline structure, thereby enhancing the barrier ability. Two-dimensional materials have been proven to be good barrier materials despite the fact that they are in the form of a single- or few-atomic layers. In particular, 2D heterostructures holds great potential for barrier application as in some cases the resistance of some 2D materials, such as MoS_2_, h-BN and WS_2_, are too high for the direct ED of Cu and the layered arrangement can remedy the surface imperfection of individual layer. SAMs are flexible enough to cover the defects and stepedges of the substrate surface and can effectively prevent Cu diffusion. Current research has been focusing on the electroless method for the attachment of Cu to SAMs, as SAMs are not conductive enough for direct electroplating. However, ED of Cu on SAMs can possibly be realized with the help of conductive SAMs or by pre-depositing metal seeds on the top of SAMs. Mixing diverse elements with different sizes can increase configurational entropy, cause large lattice distortion and suppress the formation of the crystalline structure. Due to the high thermal and mechanical stability of HEAs and HEA-N, they have been proven to be efficient barrier materials for restraining Cu penetration at high temperatures.

Although plenty of work has been conducted on the development of credible barrier materials, there remain some unsolved issues such as the poor quality of Ru thin film grown via PVD or CVD and the defects and stepedges on 2D materials formed in manufacturing. To be specific, the growth of Ru thin films via PVD or CVD often leads to a columnar structure and grain boundaries, which are vulnerable to Cu diffusion at elevated temperatures. Therefore, there is a pressing need to develop Ru manufacturing protocols for the production of high-quality thin film of Ru. Electrochemical deposition of a Ru barrier layer is of particular attraction due to the formation of a conformal thin film with a well-ordered structure. To amend their imperfection caused in manufacturing, the incorporation of 2D materials with SAMs is a good option, as SAMs can preferentially cover the defective areas on 2D materials such as pinholes or stepedges.

## Figures and Tables

**Table 1:** Brief comparison of properties, fabrication methods and expected thickness of barriers.

Barriers	Resistivity (μΩ·cm)	Melting Point (°C)	Deposition Method	Expected Thickness
Ta/TaN	Ta > 13	Ta ~ 2996	PVD or CVD	A few nm
PGMs	Ru ~ 7 Ir ~ 4.7	Ru ~ 2334 Ir ~ 2454	PVD, CVD, ALD, ED, electroless deposition	Few nm
2D materials	Graphene ~ 1	Graphene ~ 3652	CVD	~1 nm
SAMs	/	/	Solution immersion	Monolayer
HEAs	Poor	Normally > 1000	Magnetron sputtering, laser cladding, ED, electron beam evaporation	Few nm

**Table 2:** Microstructure, lattice constants and hardness. Reproduced from Tung et al. [180]. Copyright 2006 Elsevier B.V. All rights reserved.

Alloys	Microstructure	FCC Lattice Constants (Å)	BCC Lattice Constants (Å)	Hardness (HV)
AlCoCrCuFeNi	FCC+BCC	3.60	2.87	420
Al_0.5_CoCrCuFeNi	FCC	3.59	-	208
AlCo_0.5_CrCuFeNi	FCC+BCC	3.62	2.87	473
AlCoCr_0.5_CuFeNi	FCC+BCC	3.61	2.87	367
AlCoCrCu_0.5_FeNi	BCC	-	2.87	458
AlCoCrCuFe_0.5_Ni	FCC+BCC	3.61	2.87	418
AlCoCrCuFeNi_0.5_	FCC+BCC	3.63	2.87	423
